# A Prediction Model on Viscoelastic Fatigue Damage of Asphalt Mixture

**DOI:** 10.3390/ma13173782

**Published:** 2020-08-27

**Authors:** Limin Li, Xiaoling Jiang, Yuliang Lin, Heng Yan

**Affiliations:** 1 School of Civil and Environmental Engineering, Hunan University of Science and Engineering, Yongzhou 425199, China; jl2015@126.com; 2 School of Civil Engineering, Central South University, Changsha 410075, China; linyuliang11@csu.edu.cn; 3 School of Civil Engineering, Chongqing Jiaotong University, Chongqing 400074, China; c2015ch@126.com

**Keywords:** asphalt mixture, viscoelasticity, fatigue damage, prediction model

## Abstract

Fatigue damage affects both durability and safety, and it has been the most important distress in asphalt concrete. Fatigue damage occurs as a result of repeated traffic loading. An asphalt mixture is a typical viscoelastic material, and its fatigue damage is related to its viscoelastic properties. Under repeated traffic loading, the combined effects of creep damage and fatigue damage will shorten its fatigue life. Currently, the evaluation of the fatigue damage of asphalt mixtures rarely considers the combined effects of creep damage and fatigue damage. To solve this problem, a viscoelastic fatigue damage prediction model of an asphalt mixture considering the combined effects of creep damage and fatigue damage is put forward by introducing parameter β and a displacement factor based on theoretical derivations and testing. The results show that the model can embody the viscoelastic fatigue damage essence of asphalt mixtures, and it can also consider the effects of aging degree, temperature, load frequency and stress on fatigue damage of asphalt mixtures. The maximum relative error of the testing and prediction results of fatigue life is 0.15, and it is a reasonable prediction model.

## 1. Introduction

Under repeated traffic loading, the creep and fatigue cracking of asphalt pavement will occur, which will eventually cause fatigue damage of the asphalt pavement. Fatigue damage in asphalt pavements propagates due to repetitive vehicular loads. To solve the fatigue damage problem of asphalt pavements, a lot of research on fatigue damage of asphalt mixtures has been performed, based on damage mechanics. El-Basyouny et al. [1] put forward the fatigue model of the 2002 design guide (DG) model based on the Asphalt Institute MS-1 model. Based on the uniaxial constant strain rate tests, a viscodamage (VD) model that could reasonably predict the fatigue damage of asphalt was given by Masoud et al. [2]. Adhikari et al. [3] compared the abilities of different models to predict fatigue life on the basis of Rowe and Bouldin’s stiffness degradation concept, and found that the energy-based fatigue prediction model correlated well with the laboratory-determined fatigue life. Pronk et al. [4,5] found that the Asphalt Concrete Pavement-Fatigue (ACP-F) model was very good for fitting the evolution of a measured complex stiffness modulus for the whole specimen during a 4PB fatigue test, and reported the processing of four point bending test results for viscoelasticity and fatigue models. Wei et al. [6] proposed a new parameter of material fatigue sensitivity representing asphalt mixtures’ fatigue resistance by using the viscoelastic continuum damage approach. The fatigue damage characteristics of asphalt mixtures were analyzed by Kim et al. [7] using the viscoelastic continuous damage model. A practical method predicting fatigue behavior of the asphalt mixture was put forward by Mello et al. [8], based on the bending fatigue test and the continuous damage theory. The study of fatigue damage characteristics in asphalt mixtures was studied by Ni et al. [9] using the damage variable defined by residual strength decay, and it was found that the fatigue damage model could reflect the damage state of asphalt mixtures more accurately. Mahmoud et al. [10] applied viscoelastic continuum damage theory to simulate the asphalt fatigue properties, and found that there was a good correlation between viscoelastic parameters and anti-fatigue performance of asphalt mixture. Liu et al. [11] found that the residual strength-dynamic modulus coupled model could better describe the fatigue damage evolution law of an asphalt mixture, and the parameter of this coupled model could be obtained by fewer residual strength tests. The fatigue life model of an asphalt mixture based on the definition of damage variable was put forward by Castro et al. [12]. Zhu et al. [13] considered that the structural failure of an asphalt mixture was caused by the gradual accumulation of micro-cracking due to the comprehensive damage of fatigue and creep. Meanwhile, the damage model of interaction between fatigue and creep for asphalt mixtures was proposed, but it lacked further verification. Zheng et al. [14] put forward the nonlinear fatigue damage model for asphalt mixtures based on the elementary theory of fatigue damage. Sun et al. [15] found that the elasticity-power hardening model and the Sidoroff damage model could be used to describe the constitutive relation of damage failure stage. Guan et al. [16,17,18,19,20] reported the fatigue life prediction equation and damage evaluation model of asphalt mixtures. Zhang et al. [21] found that the damage effect would occur in the creep process of asphalt mixtures. An asphalt mixture is a viscoelastic material, and its fatigue performance is related to time, temperature and stress. Considering the creep effect, the viscoelastic fatigue damage model of asphalt mixtures has still been rarely addressed at present. Therefore, to solve the fatigue damage problem of asphalt pavement, it is necessary to investigate the prediction model on viscoelastic fatigue damage of an asphalt mixture considering the creep effect.

## 2. Model on Viscoelastic Fatigue Damage of Asphalt Mixture and Its Parameters

### 2.1. Model on Viscoelastic Fatigue Damage of Asphalt Mixture

According to the literature [22], the damage variable can be defined as Equation (1).

(1) $$D=\frac{A-\tilde{A}}{A}$$

where D is the damage variable, A is the area without damage and $\tilde{A}$ is the effective load bearing area.

On the basis of literature [23], for the trabecular fatigue test, the damage variable D and the damage increment ΔD could be calculated as follows:

(2) $$D=1-\frac{S_{\left(N\right)}}{S_{(0}{}_{)}}$$

(3) $$\Delta D=1-\frac{S_{N+1}}{S_{N}}$$

where $S_{\left(0\right)}$ is the initial stiffness modulus, $S_{\left(N\right)}$ is the stiffness modulus after the cyclic loading of N times and $S_{N}$ and $S_{N+1}$ are the stiffness moduli before and after the cyclic load action of unit times respectively. Based on the damage increment evolution law under different load times, taking 100 times as the unit times of the cyclic load action, the critical failure point is the interface between the stable stage and the accelerated failure stage of the ΔD-N curve, and the number of loading actions of the critical failure point is the fatigue life.

The fatigue damage of an asphalt mixture will decrease under the action of vehicle load. For the stress fatigue test of σ(t)=σsinωt, its strain can be expressed as Equation (4).

(4) $$\varepsilon (t)=\sigma [J_{1}(\omega )\sin\omega t-J_{2}(\omega )\cos\omega t]=\left|J^{*}\right|\sigma (\cos\omega t\sin\varphi +\sin\omega t\cos\varphi )\cos\omega t$$

where $\left|J^{*}\right|$, $J_{1}(\omega )$ and $J_{2}(\omega )$ are creep compliance, storage compliance and dissipation compliance, respectively, and ω, σ and φ are angular velocity, stress amplitude and phase angle, respectively [24]. Let creep compliance vary linearly in a cycle [25]. Then the creep compliance of the nth cycle at time t can be obtained according to Equation (5).

(5) $$\left|J^{*}\right|=\left|J_{n-1}^{*}\right|+\frac{\left|J_{n}^{*}\right|+\left|J_{n-1}^{*}\right|}{{T^{\prime }}_{n}-{T^{\prime }}_{n-1}}(t-{T^{\prime }}_{n-1})$$

The energy dissipation rate in a cycle is

(6) $$\Delta W_{n}=\int_{T_{n-1}}^{T_{n}}\varepsilon (t)d\sigma (t)=\int_{T_{n-1}}^{T_{n}}\left|J^{*}\right|\sigma^{2}\omega (\sin\omega t\cos\omega t\cos\varphi +\cos^{2}\omega t\sin\varphi )dt$$

Substituting Equation (5) into the energy dissipation rate Equation (6), the results shown in Equation (7) can be obtained.

(7) $$\begin{array}{l} \Delta W_{n}=\int_{{T^{\prime }}_{n-1}}^{T_{n}{}^{\prime }}[\frac{\left|J_{n-1}^{*}\right|{T^{\prime }}_{n}-\left|J_{n}^{*}\right|{T^{\prime }}_{n-1}}{{T^{\prime }}_{n}-{T^{\prime }}_{n-1}}+\frac{\left|J_{n}^{*}\right|-\left|J_{n-1}^{*}\right|}{{T^{\prime }}_{n}-{T^{\prime }}_{n-1}}t]\sigma^{2}\omega \sin\omega t\cos\omega t\cos\varphi dt+\\ \int_{{T^{\prime }}_{n-1}}^{{T^{\prime }}_{n}}[\frac{\left|J_{n-1}^{*}\right|{T^{\prime }}_{n}-\left|J_{n}^{*}\right|{T^{\prime }}_{n-1}}{{T^{\prime }}_{n}-{T^{\prime }}_{n-1}}+\frac{\left|J_{n}^{*}\right|-\left|J_{n-1}^{*}\right|}{{T^{\prime }}_{n}-{T^{\prime }}_{n-1}}t]\sigma^{2}\omega \sin\varphi \frac{1+\cos2\omega t}{2}dt\\ =-\frac{\sigma^{2}\omega \sin\varphi (\left|J_{n}^{*}\right|-\left|J_{n-1}^{*}\right|)}{4}+\frac{\sigma^{2}\omega \sin\varphi }{2}[\frac{1}{2}(\left|J_{n}^{*}\right|T^{\prime }+\left|J_{n-1}^{*}\right|T^{\prime })]\\ \;=\frac{\sigma^{2}}{4}(J_{1_{n-1}}-J_{1_{n}})+\pi \sigma^{2}\sin\varphi \left|{\bar{J}}_{n}^{*}\right|\\ \;=\frac{\sigma^{2}}{4}(J_{1_{n-1}}-J_{1_{n}})+\pi \sigma^{2}{\bar{J}}_{2_{n}}\; \end{array}$$

where t is the load action time, ${T^{\prime }}_{n-1}$ is the total time of *n* − 1 periods, $T_{n}{}^{\prime }$ is the total time of *n* periods, *T* is the total time of one period, $J_{1_{n-1}}$ is storage compliance at the end of ${T^{\prime }}_{n-1}$, $J_{1n}$ is storage compliance at the end of $T_{n}{}^{\prime }$, $\left|J_{n}^{*}\right|$ is the average value of the creep compliance in the range of ${T^{\prime }}_{n-1}$ to $T_{n}{}^{\prime }$ and ${\bar{J}}_{2n}$ is the average value of the dissipation compliance in the range of ${T^{\prime }}_{n-1}$ to $T_{n}{}^{\prime }$.

For an asphalt mixture, when its fatigue damage occurs, compared with the viscous energy consumption, there is no energy consumption of elasticity. Therefore, the cumulative dissipation energy can be obtained according to Equation (8).

(8) $$W_{N}=\sum_{n=1}^{N}\Delta W_{n}=\sum_{n=1}^{N}\frac{\sigma^{2}}{4}(J_{1_{n-1}}-J_{1_{n}})+\pi \sigma^{2}{\bar{J}}_{2_{n}}=\sum_{n=1}^{N}\pi \sigma^{2}{\bar{J}}_{2_{n}}$$

The damage variable of *D* is introduced, and based on the assumption of strain equivalence, Equation (9) can be obtained.

(9) $$\tilde{\sigma }=\frac{\sigma }{1-D}$$

where σ is Cauchy stress and $\tilde{\sigma }$ is effective stress. Substitution of Equation (9) into the cumulative dissipation energy Equation (8) results in Equation (10).

(10) $$W_{N}=\sum_{n=1}^{N}\frac{\pi \sigma^{2}J_{2}(\omega )}{{\left(1-D_{n}\right)}^{2}}$$

Viscoelastic materials subjected to repeated stresses can be expressed as Equation (11).

(11) $$\sigma (t)=\sigma_{0}e^{i\omega t}$$

The strain response is shown in Equation (12).

(12) $$\varepsilon (t)=\varepsilon^{*}e^{i\omega t}=\varepsilon_{0}e^{i(\omega t-\delta )}$$

Substitution of Equations (11) and (12) into the differential relation Pσ=Qε results in Equation (13).

(13) $$\varepsilon (t)/\sigma (t)=\varepsilon^{*}/\sigma_{0}=J^{*}(i\omega )=J_{1}(\omega )-iJ_{2}(\omega )$$

where $J^{*}(i\omega )$, $J_{1}(\omega )$ and $J_{2}(\omega )$ are dynamic compliance, storage compliance and dissipation compliance, respectively.

For the stress fatigue test of σ(t)=σsinωt, its strain response can be expressed as Equation (14).

(14) $$\varepsilon^{*}(t)=\frac{\sigma [J_{1}(\omega )\sin\omega t-J_{2}(\omega )\cos\omega t]}{1-D}$$

The energy consumption of 1th periods is

(15) $$\begin{array}{l} W=\int_{0}^{T}\sigma (t)d\varepsilon_{t}^{*}(t)=\int_{0}^{T}\sigma \sin\omega t\{\sigma \omega [J_{1}(\omega )\cos\omega t+J_{2}(\omega )\sin\omega t]{\left(1-D\right)}^{-1}\}dt\\ \;=\pi \sigma^{2}J_{2}(\omega )/(1-D) \end{array}$$

Then the accumulated dissipated energy from star to nth periods can be obtained according to Equation (16).

(16) $$W_{N}=\sum_{n=1}^{N}\frac{\pi \sigma^{2}J_{2}(\omega )}{{\left(1-D_{n}\right)}^{2}}$$

According to the equivalence principle of time and temperature [26], when the temperature changes from T to $T_{0}$, there is

(17) $$J(t,T)=J(t/\alpha_{T},T_{0})$$

(18) $$W_{N}=\sum_{n=1}^{N}\frac{\pi \sigma^{2}J_{2}(\alpha_{T}\omega )}{{\left(1-D_{n}\right)}^{2}}$$

where $\alpha_{\tau }$ is shift factor. When the temperature is $T_{0}$, $\mathrm{lg}\alpha_{T}=0$, the shift factor $\alpha_{\tau }$ is calculated by the Williams–Lendel–Ferry (WLF) equation, and it can be expressed in Equation (19).

(19) $$\mathrm{lg}\alpha_{T}=-\frac{C_{1}(T-T_{0})}{C_{2}+T-T_{0}}$$

where $C_{1}$ and $C_{2}$ are constants and $T_{0}$ is the reference temperature, in °C.

On the basis of the definition of integral summation, the expression for Equation (18) can be derived as Equation (20).

(20) $$W_{N}=\sum_{n=1}^{N}\frac{M}{{\left(1-D_{n}\right)}^{2}}=\int_{0}^{N}\frac{M}{{\left(1-D_{n}\right)}^{2}}dn$$

Based on the damage definition of dissipated energy, damage value $D_{N}$ of *N* times load action can be expressed in Equation (21).

(21) $$D_{N}=\frac{W_{N}}{W_{N_{f}}}$$

where $W_{N}$ and $W_{N_{f}}$ are the cumulative dissipated energy from star to nth periods and the cumulative dissipated energy of destruction, respectively.

The expression for its damage evolution equation can be derived as Equation (22).

(22) $$dD/dN={\left(\partial W_{N}/\partial N\right)}^{\beta }$$

where *β* is a parameter related to material aging degree, stress amplitude, loading frequency, temperature, etc. [26].

On the basis of Equations (20) and (22), Equation (23) can be obtained.

(23) $$dD/dN={\left(\partial W_{N}/\partial N\right)}^{\beta }=M^{\beta }/{\left[1-D(N)\right]}^{2}{}^{\beta }$$

On the basis of the integral of Equation (23) and the condition of D=1, $N=N_{f}$, the viscoelastic fatigue damage model of the asphalt mixture can be obtained as follows:

(24) $$D(N)=1-{\left[1-N(1+2\beta )/M^{-\beta }\right]}^{\frac{1}{1+2\beta }}=1-{\left(1-\frac{N}{N_{f}}\right)}^{\frac{1}{1+2\beta }}$$

(25) $$N_{f}=M^{-\beta }/(1+2\beta )={\left[\pi \sigma^{2}J_{2}(\alpha_{T}\omega )\right]}^{-\beta }/(1+2\beta )$$

where $N_{f}$ is fatigue life.

### 2.2. Model Parameters

**Figure 1:**
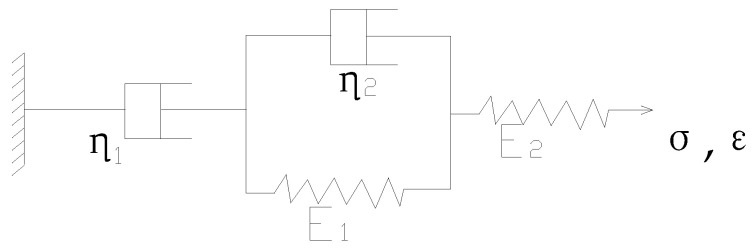
Burgers’ model.

Based on the existing research results [16,27,28,29], Burgers’ viscoelastic model shown in Figure 1 is used. The dissipation compliance $J_{2}(\alpha_{T}\omega )$ is then

(26) $$J_{2}(\alpha_{T}\omega )=\frac{1}{\eta_{2}\alpha_{T}\omega }+\frac{\eta_{2}\alpha_{T}\omega }{E_{2}^{2}+\eta_{2}^{2}{\left(\alpha_{T}\omega \right)}^{2}}$$

with

(27) $$\eta_{1}\dot{\varepsilon }+\frac{\eta_{1}\eta_{2}}{E_{3}}\ddot{\varepsilon }=\sigma +(\frac{\eta_{1}}{E_{1}}+\frac{\eta_{1}+\eta_{2}}{E_{3}})\dot{\sigma }+\frac{\eta_{1}\eta_{2}}{E_{1}E_{2}}\ddot{\sigma }$$

For constant stress $\sigma_{0}$, there is

(28) $$\varepsilon (t)=\sigma_{0}[\frac{1}{E_{1}}+\frac{t}{\eta_{1}}+\frac{1}{E_{2}}(1-e^{-tE_{2}/\eta_{2}})]$$

According to $\varepsilon (t)=\sigma_{0}J(t)$, the creep compliance can be obtained as follows:

(29) $$J(t)=\frac{1}{E_{1}}+\frac{t}{\eta_{1}}+\frac{1}{E_{2}}(1-e^{-tE_{2}/\eta_{2}})$$

For rectangular wave uniaxial dynamic load creep tests with period *T*, there is

(30) $$\sigma_{t}=\left\{ \begin{array}{l} \sigma_{0\;},\;0\leq t\leq t_{0}\\ 0\;,\;0<t\leq T\; \end{array}\right.$$

where $\sigma_{0}$ is the axial stress and $t_{0}$ is load action time in one period *T*. Under the stress $\sigma (t)=\sigma_{0}H(t)$ action, the strain of viscoelastic material is

(31) $$\varepsilon (t)=J(t)\sigma_{0}$$

The variable load can be regarded as the superposition of multiple forces. The expression for the strain generated by the additional stress $\Delta \sigma_{1}$ at time $\zeta_{1}$ can be derived as Equation (32).

(32) $$\Delta \varepsilon_{1}=J(t-\zeta_{1})\Delta \sigma_{1}$$

Then at time *t* after the time $\zeta_{1}$, the value of strain generated by $\sigma_{0}$ and $\Delta \sigma_{1}$ together can be obtained as follows:

(33) $$\varepsilon (t)=\sigma_{0}J(t)+\Delta \sigma_{1}J(t-\zeta_{1})$$

**Figure 2:**
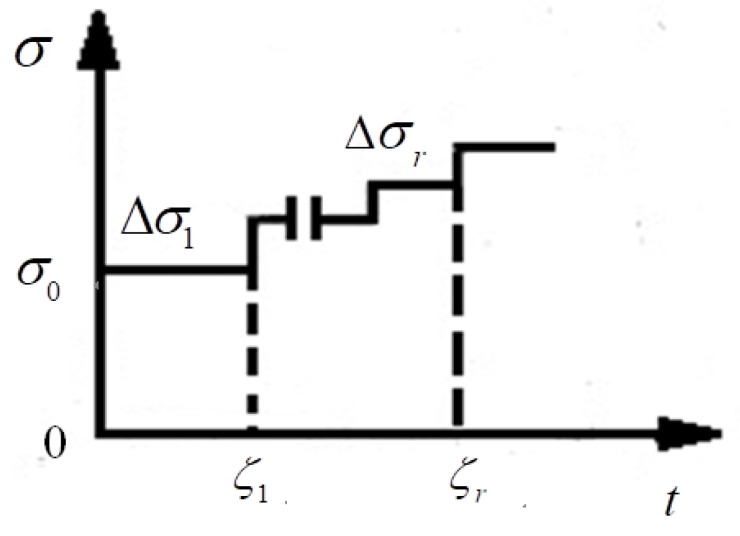
Boltzmann’s superposition principles.

Based on Boltzmann’s superposition principles shown in Figure 2, the total strain of time *t* after the time $\zeta_{r}$ is

(34) $$\varepsilon (t)=\sigma_{0}J(t)+\sum_{i=1}^{r}\Delta \sigma_{i}J(t-\zeta_{i})$$

If σ(t) is a continuous differentiable function, it can be decomposed into stress $\sigma_{0}H(t)$ and a lot of small stresses dσ(ζ)⋅H(t−ζ). Based on Boltzmann’s superposition principles, Equation (35) can be obtained.

(35) $$\varepsilon (t)=\sigma_{0}J(t)+\int_{0}^{t}\sigma (\varsigma )\frac{dJ(t-\zeta )}{d(t-\zeta )}d\zeta$$

In the same way, on the basis of Boltzmann’s superposition principles, the strain at time *t* after unloading can be written as Equation (36).

(36) $$\varepsilon (t)=\sigma_{0}J(t)-\sigma_{0}J(t-t_{0})$$

On the basis of Equations (26) and (36), Equation (37) can be obtained.

(37) $$\varepsilon (t)=\frac{\sigma_{0}t_{0}}{\eta_{1}}+\frac{\sigma_{0}}{E_{2}}(e^{E_{2}t_{0}/\eta_{2}}-1)e^{-E_{2}t/\eta_{2}}$$

Let the residual strain of the ith cycle gap load at time (N−i)T to the residual strain after the *N*th load be $\varepsilon_{Ni}$. According to Equation (37), Equation (38) can be obtained.

(38) $$\varepsilon_{Ni}=\frac{\sigma_{0}t_{0}}{\eta_{1}}+\frac{\sigma_{0}}{E_{2}}(e^{E_{2}t_{0}/\eta_{2}}-1)e^{-E_{2}(N-i)/\eta_{2}}$$

The sum of residual strains generated by *N* times load action is the cumulative residual strain generated by *N* times load action [21,30]. Based on Boltzmann’s superposition principles, the cumulative residual strain $\varepsilon_{N}$ can be obtained as follows:

(39) $$\varepsilon_{N}=\sum_{i=1}^{N}\varepsilon_{Ni}=\frac{\sigma_{0}t_{0}}{\eta_{1}}N+\frac{\sigma_{0}(e^{E_{2}t_{0}/\eta_{2}}-1)e^{E_{2}T/\eta_{2}}}{E_{2}(1-e^{E_{2}T/\eta_{2}})}(1-e^{-E_{2}NT/\eta_{2}})$$

where $\eta_{2}$ and $E_{2}$ are the viscoelastic parameters of Burgers’ model, and they can be obtained using nonlinear curve fitting based on the creep test datum of the asphalt mixture and Equation (39).

The dynamic creep tests of the asphalt mixture at different temperatures can be carried out. On the basis of the reference temperature $T_{0}=25{\;}^{\circ }C$ and test data, with the load action time t and creep compliance taken as logarithmic coordinates, the $\mathrm{lg}\alpha_{T}$ of different temperatures can be obtained using the horizontal shift function in Origin 7.0 software (OriginLab Corporation, Northampton, MA, USA). Then for the $\mathrm{lg}\alpha_{T}$ of different temperatures, according to Equation (19), $C_{1}$ and $C_{2}$ can be obtained using the linear fitting function in 1stopt 6.0 software (7D-Soft High Technology Inc., Beijing, China). Thus, shift factor $\alpha_{T}$ and dissipation compliance $J_{2}(\alpha_{T}\omega )$ can be calculated through Equations (19) and (26), respectively.

Fatigue testing and dynamic creep testing of the asphalt mixture can be conducted, and fatigue life $N_{f}$ and $M=\pi \sigma^{2}J_{2}(\alpha_{T}\omega )$ can be obtained. Then according to $N_{f}=M^{-\beta }/(1+2\beta )$, the parameter β can be obtained using the linear fitting function in 1stopt software.

## 3. Model Validation

(1) Let n=2β and *k* = ${\left[\pi J_{2}(\alpha_{T}\omega )\right]}^{-\beta }/(1+2\beta )$. According to Equation (25), we can obtain $N_{f}=k{\left(\frac{1}{\sigma }\right)}^{n}$. Therefore, the proposed fatigue damage prediction model is a simplified form of the fatigue equation used widely.

(2) According to Equation (24), Equation (40) can be obtained.

(40) $$\partial D/\partial N={\left(1-N/N_{f}\right)}^{2\beta /(1+2\beta )}/[N_{f}(1+2\beta )]>0$$

(41) $$\partial^{2}D/(\partial N\partial \sigma )>0$$

Meanwhile, when $N=N_{f}$, D=1 and when N=0, D=0. Moreover when β=0, $D=N/N_{f}$. Therefore, the proposed fatigue damage prediction model meets the physical and basic conditions of the fatigue damage function, and it can reflect the irreversibility of fatigue damage. When β=0, the damage variable *D* is consistent with the Miner linear fatigue damage variable.

(3) When β is constant, the integration of Equation (23) is performed, and Equation (42) can be obtained.

(42) $$\Delta D=1-D_{i-1}-[{\left(1-D_{i-1}\right)}^{1+2\beta }]-(1+2\beta )M^{\beta }{]}^{\frac{1}{1+2\beta }}$$

Thus, the damage variable of *D* will be written as Equation (43).

(43) $$D=\sum_{i=1}^{N}\Delta D_{i}=1-{\left[1-(1+2\beta )\sum_{i=1}^{N}M^{\beta }\right]}^{\frac{1}{1+2\beta }}$$

If fatigue failure of the asphalt mixture occurs, after $N_{1}$ times of the alternating load $F_{1}$ and $N_{2}$ times of the alternating load $F_{2}$ successively, the damage variable of *D* will be defined as

(44) $$D=\sum_{i=1}^{N_{1}+N_{2}}\Delta D_{i}=1$$

(45) $$\frac{N_{2}}{N_{f2}}={\left(1-\frac{N_{1}}{N_{f1}}\right)}^{\frac{1+2\beta_{2}}{1+2\beta_{1}}}$$

If fatigue failure of the asphalt mixture occurs, after $N_{2}$ times of the alternating load $F_{2}$ and $N_{1}$ times of the alternating load $F_{1}$ successively, the damage variable *D* will be written as Equation (46).

(46) $$D=1-{\left[{\left(1-\frac{N_{2}}{N_{f2}}\right)}^{\frac{1+2\beta_{1}}{1+2\beta_{2}}}-\frac{N_{1}}{N_{f1}}\right]}^{\frac{1}{1+2\beta_{1}}}$$

When β is constant, there is $\beta_{1}=\beta_{2}$. Thus, according to Equations (45) and (46), we can obtain D=1. Therefore, the proposed fatigue damage prediction model can reflect the nonlinear accumulation of fatigue damage and the influence of loading sequence.

(4) On the basis of the integral of the common damage evolvement model of Equation (47), the expression for the damage variable *D* can be derived as Equation (48).

(47) $$\frac{dD}{dN}={\left(\frac{\sigma }{M}\right)}^{\beta }{\left(1-D\right)}^{-(\beta +\gamma )}$$

(48) $$D=1-{\left(1-\frac{N}{N_{f}}\right)}^{\frac{1}{1+\beta +\gamma }}$$

It can be seen from Equation (48) that the damage variable *D* is similar to the proposed fatigue damage prediction model.

**Figure 3:**
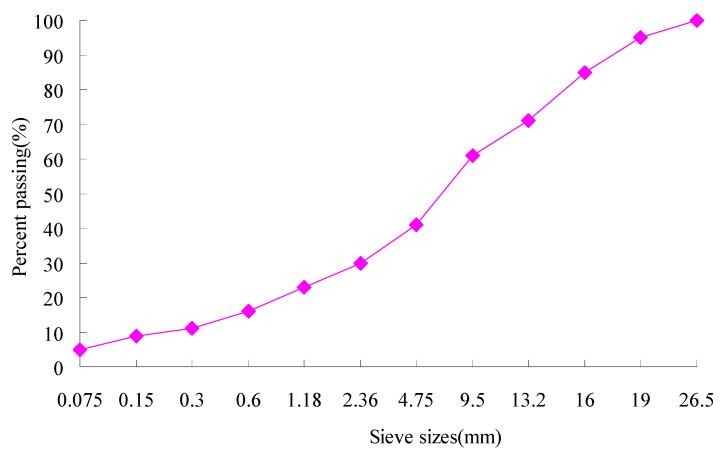
Aggregate gradation of asphalt mixture for test.

**Figure 4:**
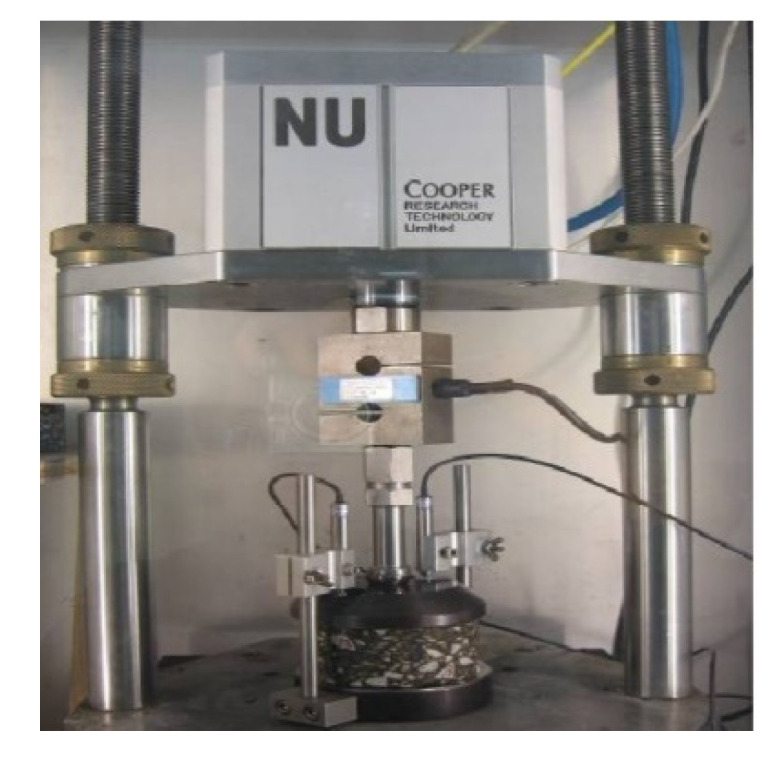
Dynamic creep test of asphalt mixture.

**Figure 5:**
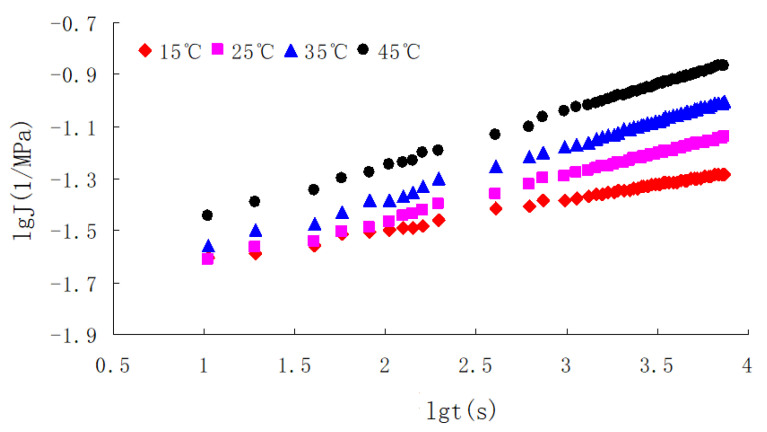
Double logarithmic curve of asphalt mixture between creep compliance and load time.

(5) Zhonghai AH-70 asphalt was used, and its properties are given in Table 1. The aggregate gradations used in the study are shown in Figure 3. The coarse aggregates and fine aggregates were limestone, and the mineral filler was crushed limestone. The properties of the coarse aggregates are listed in Table 2. The asphalt mixture AC-20 with the optimum asphalt content of 4.2% obtained by the Marshall Tests was used, and its characteristics are given in Table 3. Dynamic creep tests of the asphalt mixture were conducted by using a Cooper material testing machine from Cooper Research Technology Britain as shown in Figure 4, under the action of a rectangular wave cyclic load with the frequency of 0.5 Hz. Loading time and gap time were 1 s, and the total test time was 2 h [16,31]. The test temperatures were 15, 25, 35 and 45 °C. The load action time t and creep compliance J were taken as logarithmic coordinates, and the test results of the asphalt mixture are shown in Figure 5.

On the basis of the reference temperature $T_{0}=25^{\;\circ }C$, the $\mathrm{lg}\alpha_{T}$ of different temperatures can be obtained using the horizontal shift function in Origin software. Then for the $\mathrm{lg}\alpha_{T}$ of different temperatures, according to Equation (19), $C_{1}$ and $C_{2}$ can be obtained using the linear fitting function in 1stopt software. Thus, the expression of Equation (19) can be written as Equation (49).

(49) $$\mathrm{lg}\alpha_{T}=-\frac{4.68\times (T-25)}{-79.83+(T-25)}$$

**Figure 6:**
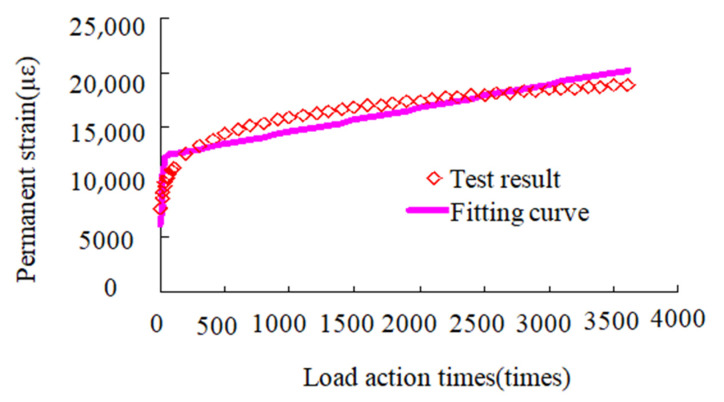
Dynamic creep test results of asphalt mixture.

According to Equation (49), the shift factor at different temperatures $\alpha_{T}$ can be obtained. Because the reference temperature is 25 °C, based on the universal global optimization method and Levenberg–Marquardt theory, according to Equation (39) and the results of the dynamic creep test of the asphalt mixture at 25 °C, the results obtained using the linear fitting function in 1stopt software are shown in Figure 6. Thus, we can obtain $E_{2}=13.04\;\mathrm{MPa}$, $\eta_{2}=262$ MPa·S.

**Figure 7:**
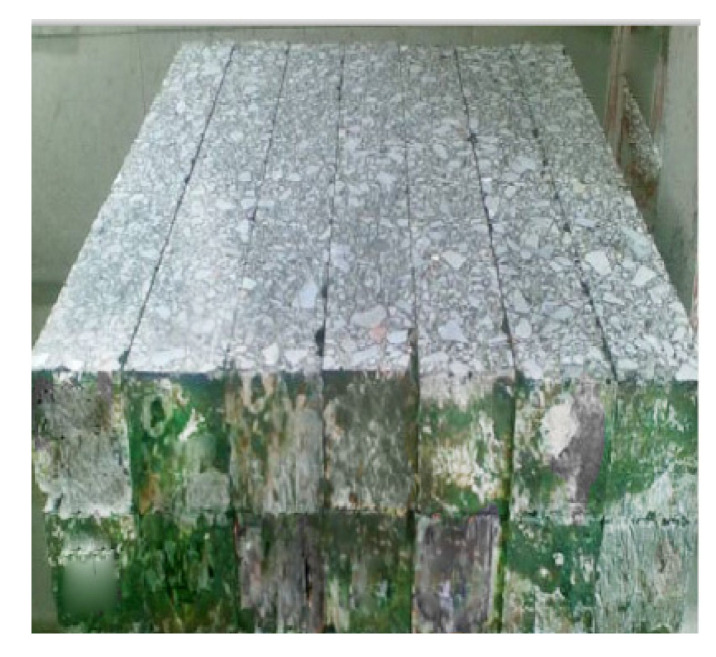
Beam samples for four point bending fatigue test.

**Figure 8:**
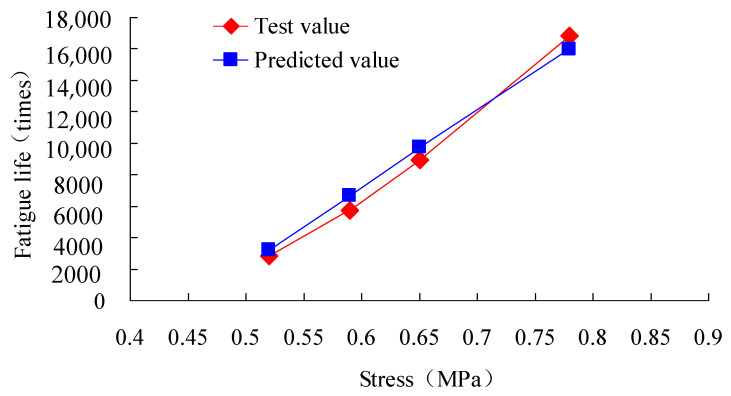
Prediction and test results of fatigue life for asphalt mixture.

Beam samples for four point bending fatigue tests are shown in Figure 7. According to JTG E20-2011 T0739-2011 [31], bending fatigue tests were performed by using a Cooper material testing machine from Cooper Research Technology Britain under the action of a half sine wave cyclic load with the frequency of 3 Hz [16]. The test temperature was 25 °C, and loading mode was control stress. Thus, we can obtain the value of $J_{2}(\alpha_{T}\omega )$, based on test results and Equation (26). Moreover, the value of M can be calculated in accordance with the formula $M=\pi \sigma^{2}J_{2}(\alpha_{T}\omega )$. Then, according to Equation (50), the result of β=1.973 can be obtained using the linear fitting function in 1stopt software, and the correlation coefficient R^2^ is 0.9923. Finally, the fatigue life of the asphalt mixture can be predicted using Equation (25). The test and prediction results of fatigue life are given in Table 4 and Figure 8, and their maximum relative error is 0.15.

(50) $$N_{f}=M^{-\beta }/(1+2\beta )$$

The above studies prove that the proposed viscoelastic fatigue model of the asphalt mixture is reasonable.

## 4. Conclusions

(1) A viscoelastic fatigue damage prediction model for asphalt mixtures considering the combined effects of creep damage and fatigue damage is proposed, using Burgers’ model, the WLF equation and Boltzmann’s superposition principles, based on bending fatigue and dynamic creep testing, and theoretic analysis and testing demonstrated that it is reasonable.
(2) The proposed model on viscoelastic fatigue damage of asphalt mixtures can consider the influences of aging, temperature, loading frequency and stress on the fatigue failure of asphalt mixtures by introducing the parameter β and shift factor, and it can embody the essence of viscoelastic fatigue damage of asphalt mixtures.
(3) To bring the model to asphalt pavement design in the future, problems such as the equivalent fatigue temperature based on fatigue damage and the field correction coefficient considering the difference between indoor testing and actual pavements should be further investigated systematically.

## Figures and Tables

**Table 1:** Asphalt properties.

Properties		Criteria	Zhonghai AH-70	Methods
Ductility at 15 °C (cm)		≥100	135	T0605-2011 [32]
Penetration degree at 25 °C (0.1 mm)		60~80	70	T0604-2011 [32]
Softening point (°C)		≥46	51	T0606-2011 [32]
Dynamic viscosity at 60 °C (Pa·s)		≥180	217	T0620-2000 [32]
After the rolling thin film oven (TFOT) (163 °C, 5 h)	Mass loss (%)	−0.36	−0.15	T0609-2011 [32]
	Ductility at 15 °C (cm)	16.2	21.2	T0605-2011 [32]
	Ductility at 10 °C (cm)	≥6	9.1	T0605-2011 [32]
	Penetration degree ratio at 25 °C (%)	−61.5	65	T0604-2011 [32]

**Table 2:** The properties of coarse aggregates.

Technical Indexes	Results	Criteria	Methods
Crush value (%)	16.4	≤26	T0316-2005 [33]
Content of acicular and flaky shape particles (%)	9.4	≤15	T0312-2005 [33]
Losses of Los Angeles Abrasion Test (%)	21.5	≤28	T0317-2005 [33]
Water absorption (%)	0.32	≤2	T0307-2005 [33]
Asphalt adhesion (graduation)	5	≥4	T0616-1993 [32]
Impact value (%)	18	≤30	T0322-2005 [33]
Firmness (%)	4.3	≤12	T0314-2005 [33]
Mud content (%)	0.7	≤1	T0310-2005 [33]

**Table 3:** Characteristics of asphalt mixture.

Optimum Asphalt Content (%)	Bulk Volume Relative Density (g_·_cm^−3^)	Volume of Air Voids (%)	Voids Filled with Asphalt (%)	Voids in Mineral Aggregate (%)	Flow Value (0.1 mm)	Marshall Stability (kN)
4.2	2.44	4.2	68.5	13.8	38.2	11.1
Specification Requirement [32]	-	3–6	55–70	>12.5	15–40	>8

**Table 4:** Test and prediction results of fatigue life.

Stress Ratio	Stress (MPa)	Value of M	Test Value of Fatigue Life (Times)	Predicted Value of Fatigue Life (Times)	Relative Error
0.4	0.52	0.00740727	2858	3227	0.13
0.45	0.59	0.00514394	5752	6627	0.15
0.5	0.65	0.00423812	8955	9711	0.08
0.6	0.78	0.00329212	16,819	15,986	−0.05
